# Activity, social and sexual behaviour in Red Junglefowl selected for divergent levels of fear of humans

**DOI:** 10.1371/journal.pone.0204303

**Published:** 2018-09-26

**Authors:** Rebecca Katajamaa, Lovisa H. Larsson, Paulina Lundberg, Ida Sörensen, Per Jensen

**Affiliations:** AVIAN Behavioural Genomics and Physiology Group, IFM Biology, Linköping University, Linköping, Sweden; University of Missouri Columbia, UNITED STATES

## Abstract

The domesticated phenotype is a set of behavioural, morphological and physiological traits that is common for domesticated species. Previous research has found that selection for tameness only can give rise to correlated selection responses that resemble the domesticated phenotype. It has therefore been suggested that tameness may drive the domesticated phenotype as correlated traits. We selected Red Junglefowl for divergent levels of fear of human for eight generations and assessed possible correlated selection responses in other behaviours in semi-natural settings. Behavioural studies were carried out on birds from generations six to eight, and at different ages, in order to study possible effects on general activity, social behaviour and male courtship behaviour. We found that the differences between selection lines changed with age. Adult low fear birds were generally more active and high fear males showed a more intense courtship behaviour. Our study shows that several behaviours can be modified through correlated selection responses by selection on reduced fear of humans only, emphasising the putative role of tameness as a driver of domestication related phenotypes.

## Introduction

Domestication is an evolutionary process that happens through many generations of genetic and phenotypic change in animal populations that live in an environment controlled by humans [1]. Animal domesticates have a set of phenotypic traits in common, usually termed the domesticated phenotype [2]. These are related to certain morphological and physiological characteristics, and importantly to behaviour, e.g., increased social tolerance, reduced activity and increased sexual promiscuity. One of the most important behavioural features of domesticated animals is that they tolerate and sometimes even actively seek human companionship. It has been suggested that the development of the domesticated phenotype is driven by this increased tameness, for example through genetic mechanisms such as pleiotropy or linkage [3].

To test this idea experimentally, a several studies have used selection for increased tameness and decreased fear of humans to assess correlated responses. Consistent with the hypothesis, selection on this behavioural trait has been accompanied by correlated selection responses in morphology, behaviour and physiology in a number of species, e.g. mink (*Neovison vison*) [4], silver fox (*Vulpes vulpes*) [5], rat (*Rattus norvegicus*) [6] and chicken (*Gallus gallus*) [7]). Some of the changes correspond to what is found in the domesticated phenotype. For example, silver foxes selected for tameness developed floppy ears and loss of pigmentation in the fur and showed behavioural changes such as whimpering for human attention and seeking human contact [5]. Rats selected for tameness tolerated handling by humans [8] and mink and chickens undergoing similar selection were also less fearful in situations involving novel arenas and objects [3, 4]. Reproduction was also affected, as shown by the fact that tame foxes became sexually mature earlier than the non-tame individuals and tended to come into heat more often [5].

In the present study, we have focused on correlated selection responses in the ancestor of the chicken, in order to model the possible early responses to domestication in an important domesticated farm species. The domestic chicken (*Gallus gallus*) originates from the Red Junglefowl, which was domesticated about 8,000 years ago in south-east Asia [9]. Red Junglefowl in the wild live in small groups of different compositions, usually one male with several females, solitary males or groups with several males [10]. This social composition is very different from the way present day domestic chickens are housed commercially. The natural habitat is another aspect that differs immensely, with Red Junglefowl living in semi-open habitats as well as forests [11]. Living in close proximity to humans is a challenge to any wild animal due to increased fear and stress, but can also convey benefits such as protection from predators and easier access to food. In a previous comparison between the Red Junglefowl and the domesticated White Leghorn, it was found that White Leghorns spend less time on foraging and general activity [12], consistent with the idea that these traits may be beneficial in the adaptation to domesticated conditions.

In order to study correlated selection responses to tameness, we selected Red Junglefowl for diverging levels of fear of human for eight generations. Birds in the Low Fear (LF) selection line were larger and produced larger offspring, and were more socially dominant compared to the High Fear (HF) birds [13]. One study also showed that LF birds had a higher metabolism as chicks while still having a better feed conversion efficiency as adults [3].

Previous studies of these selected strains of Red Junglefowl have mostly been carried out under strictly controlled experimental conditions, where the animals have been kept in small test arenas. Here, we expanded the studies to include the behaviour of the birds when kept under less intense and outdoor conditions, which may reflect the natural behaviour of the selection lines better. The aim of this study was to investigate general activity, social and sexual behaviour in Red Junglefowl of different ages selected for divergent levels of fear towards humans.

## Material and methods–General

### Ethical note

All experimental protocols were approved by Linköping Council for Ethical Licencing of Animal Experiments, ethical permit no 50–13. Experiments were carried out in accordance with the approved guidelines.

### Animals and breeding

We used Red Junglefowl that had been selected for divergent levels of fear of humans in a standardised test for six (S6), seven (S7) or eight generations (S8). Initially, they originated from two zoo populations; Copenhagen zoo (Cop) and Götala research station (Got). The Cop and Got populations were interbred for two generations to create an outbred parental generation. This was then used to select the subsequent generations of birds with divergent levels of fear of humans.

In order to carry out the selection, a standardised fear of human test was done in each generation when the birds were 12 weeks old. The birds were individually tested in a longitudinal arena in which the reaction to an approaching human was recorded. After the first outbred parental generation, selection was done within each selection group (high or low fear of humans) and the most fearful from the High Fear strain as well as the least fearful individuals from the Low Fear strain went on to be used as breeders for the next generation. For more information on the breeding scheme and selection as well as a detailed description of the Fear of Human Test, see Agnvall et al. [7]. For the remainder of the paper, birds from the High Fear group will be referred to as HF and birds from Low Fear group as LF.

### Housing conditions

Immediately after hatching, the birds were wing tagged, weighed and vaccinated against Marek’s disease. For the first five weeks, the birds were kept in small floor pens measuring 0.75 x 0.75 m during week 1–2 and 1.5 x 1.5 m for week 3–5. The pens were furnished with a heat lamp and wood chips on the floor. At five weeks of age, the animals were moved to the chicken research facility “Wood-Gush”, situated 15 km away from the hatchery. There they were housed in sex separated groups in three-level aviary pens measuring 3 x 3 x 3 m, furnished with nests, perches and wood chips as floor substrate and during periods access to an outdoor area of the same size as the pen. Room temperature was maintained around 20°C. *Ad libitum* access to feed and water was provided in both facilities as well as a 12 h light period each day with intensity of about 10 Lux.

### General procedure and ethogram

We performed three different experiments with birds of different ages, in order to study activity and social behaviour in both young and adult animals, and sexual behaviour in adult males. The birds in the different experiments were from different generations. Details about animals included and experimental procedures are given below, in association with descriptions of each of the three experiments.

Behaviours recorded in all three experiments are summarised in one single ethogram (Table 1).

**Table 1:** Ethogram of recorded behaviours.

Behaviour	Description
Feeder peck^1^	Bird pecks at feeder
Waltzing^3^	Male turns body towards female, lowers one wing with stepping movement
**Exploration**^1^^,^^**2**^	
Explore ground	Walking or standing, head close to ground, eyes focusing on ground items
Ground peck^3^	Pecks at items on ground
Object peck	Pecks at object of interest
Manipulate object	Uses beak to lift, move or otherwise manipulate object
**Feather preening**^1^^,^^**2**^	
Preening	Uses beak to trim/arrange feathers
Scratch body	Uses feet to scratch/clean/preen feathers
**Locomotion**^1^^,^^**2**^	
Walk	Two or more steps, attending to surroundings
Run	Two or more steps in faster tempo than walk, head held forward
**Vocalisation**^**2**^	
Tid-bit call	Short clucks, emitted when finding food, or by male for attracting females
Other vocalisation	Any unspecified vocalisation, not cockerel crowing
**Agonistic behaviour**^**2**^	
Wing flap threat	Bird flaps wings < 0.5 m in front of other birds, upright body position
Give and receive threat	Bird follows other bird with head high, other bird moving away
Raised hackle threat	Body horizontal or in pecking position, head towards opponent, hackles raised
Chase	Bird follows another bird, both running, jumping or flying
Attack	Bird moves swiftly towards opponent to give or gives aggressive peck. Head over opponent
Fight	Bird involved in aggressive encounter, more than one peck. Both birds active, running, jumping or flying

Behaviour categories used for statistical analysis are in bold.

^1,2,3^Behaviours or behaviour categories used in experiment 1, 2 and 3 respectively

## Experiment 1—Activity and social behaviour in young chicks

### Material and methods

The purpose of the study was to quantify differences in activity levels and social behaviour of young birds from the two selection lines while they were kept in pairs in enriched test arenas. At 5 weeks of age, birds from the eighth selected generation (20 LF [15 female, 5 male], 32 HF [18 female, 14 male]) were tested in an arena (117 x 80, height 119 cm) furnished with woodchips, a water- and a feeding bell, perches, dark nesting box and a small pile of hay with no visual contact with humans. The birds were brought to the arena at 17:00 in the evening the night before the test to habituate and the lights were left on for two hours. The following morning the lights were turned on at the start of the test session, which was at 06:00. Behaviour (Table 1) recordings were done for 10 hours from 06:00 in the morning until 16:00 in the afternoon.

Each arena contained two birds from the same selection group (HF or LF) on each test day. When possible, we used one male and one female, but some groups contained two females since the access to males was limited. In total, we tested 16 pairs from HF (of which 4 same-sex) and 9 pairs from LF (of which 4 same-sex). Behaviours were scored continuously using The Observer® XT version 12 (Noldus) for 15 continuous minutes per recorded hour, in total 150 minutes for each bird, equally distributed over the day. The behaviours were recorded as total time in seconds. Differences between selection groups were tested statistically with Generalized Linear Models (GLM) using SPSS version 24, using the pairs as independent replicates. Selection line (HF or LF), sex (male or female) and their interaction were used as predictors. For the behaviours feather preening and locomotion, scale response ‘linear’ with link function ‘identity’ was used. Gamma distribution with log link was used for behaviours feeder peck and exploration. The Wald χ^2^-statistics for GLMs was used for determining significance. Cramer’s V was calculated as a measure of effect size.

### Results

**Fig 1:**
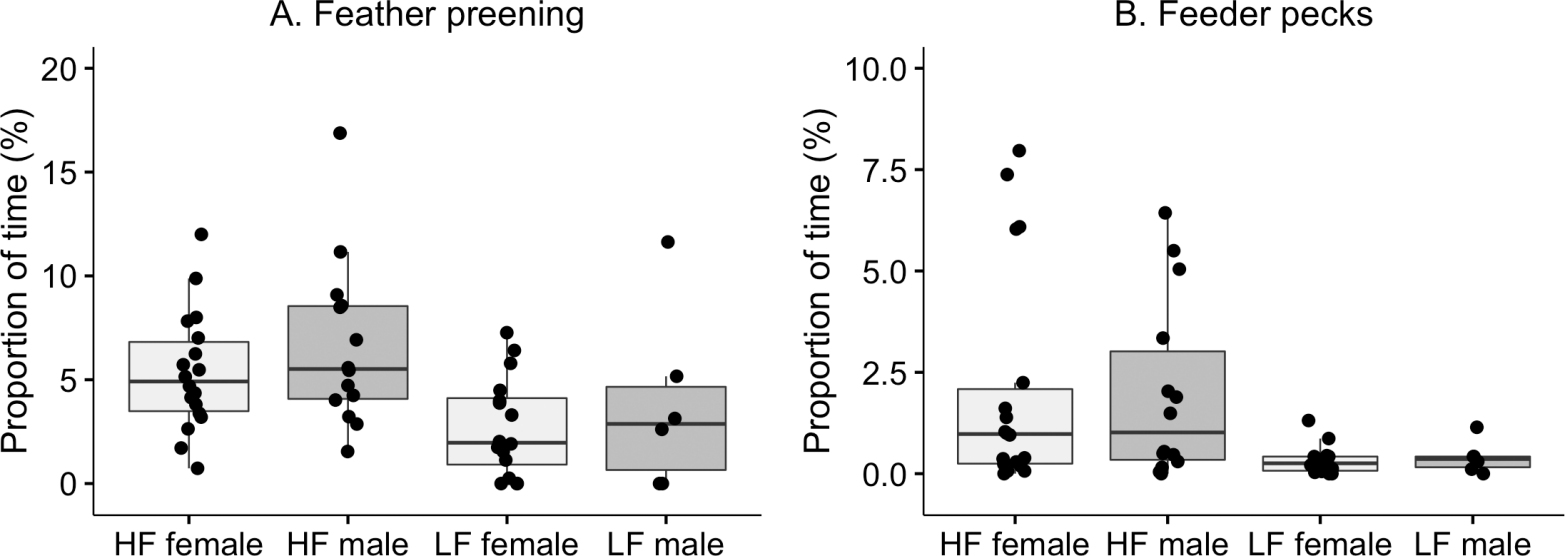
Feather preening and feeder peck. Box- and scatterplots of total duration of feather preening (A) and feeder peck (B). Presented as proportion of total time (%) in five weeks old Red Junglefowl. Each dot represents the value for one individual in each pair. The horizontal lines show median values, the boxes show the upper and lower quartiles, and the vertical lines show the minimum and maximum values, excluding outliers.

For two of the recorded behaviours there was a significant selection effect (see Table 2 for the outcome of the statistical tests of all behaviours). There was a significant selection effect on time spent feather preening (Fig 1A; V = 0.32) where LF birds spent less time on the behaviour. We did not find any effects of sex or interaction effects. An effect of selection was also found on the amount of feeder pecks (Fig 1B; V = 0.62), again with LF birds pecking less at the feeders. No effects of sex or interaction were observed. No significant selection effect was found on the time spent on locomotion or exploration (Table 2). An effect of sex was found on exploration (Table 2; V = 0.38) where females spent more time on the behaviour. Table 3 provides descriptive data for behaviours that are not presented in graphs.

**Table 2:** P-values and test statistics for all recorded behaviours.

	P-value (test statistic)		
Behaviour	Selection	Sex	Interaction
Feather preening^1^	0.020 (χ^2^ = 5.428)	0.138 (χ^2^ = 2.204)	0.878 (χ^2^ = 0.024)
Feeder pecks^1^	0.000 (χ^2^ = 19.453)	0.678 (χ^2^ = 0.172)	0.625 (χ^2^ = 0.239)
Locomotion^1^	0.931 (χ^2^ = 0.007)	0.445 (χ^2^ = 0.584)	0.386 (χ^2^ = 0.753)
Exploration^1^	0.966 (χ^2^ = 0.002)	0.011 (χ^2^ = 6.509	0.413 (χ^2^ = 0.671)
Locomotion^2^	<0.001 (χ^2^ = 15.181)	0.811 (χ^2^ = 0.057)	0.349 (χ^2^ = 0.877)
Feather preening^2^	0.113 (χ^2^ = 2.517)	0.230 (χ^2^ = 1.438)	0.001 (χ^2^ = 10.462)
Exploration^2^	0.006 (χ^2^ = 7.611)	0.004 (χ^2^ = 8.105)	0.529 (χ^2^ = 0.395)
Vocalisation^2^	<0.001 (χ^2^ = 22.307)	0.834 (χ^2^ = 0.044)	0.932 (χ^2^ = 0.007)
Agonistic social behaviours^2^	0.158 (χ^2^ = 1.998)	0.001 (χ^2^ = 10.810)	-
Ground pecks^3^	0.016 (U = 1)	-	-
Waltzing^3^	0.463 (U = 9)	-	-
Close zone^3^	0.917 (U = 12)	-	-

^1, 2^Behaviours recorded in experiment 1, and 2 respectively, analysed with generalised linear model.

^3^Behaviours recorded in experiment 3, analysed with Mann-Whitney U-test.

**Table 3:** Medians and interquartile range (IQR).

	Median (IQR) % of time	
Behaviour	High fear	Low fear
Locomotion^1^	1.5 (0.83–1.83)	1.4 (0.91–1.92)
Exploration^1^	3.8 (1.62–5.90)	4.2 (3.93–6.63)
Agonistic social behaviours^2^	0.1 (0.00–0.73)	0.3 (0.20–0.63)
Waltzing^3^	2.7 (2.67–3.00)	1.7 (1.33–2.33)
Close zone^3^	90.3 (83.67–94.00)	89.7 (72.00–95.67)

Medians and interquartile range for behaviours that have not been presented in graphs.

^1, 2, 3^Behaviours recorded in experiment 1, 2 and 3 respectively.

### Discussion

Our results showed some correlated effects of tameness on other behaviour in young Red Junglefowl. Birds selected for low levels of fear of humans spent less time on feather preening and feeder pecks. Effects of general activity measured through time spent on locomotion and exploration were not seen, although females spent more time on exploration compared to males.

The relationship between general activity level and domestication has been investigated in earlier experiments. White Leghorn layers that have been selected for intense production spend less time on active behaviours and exploration compared both to Red Junglefowl and to domesticated birds from an ancestral breed, the Swedish bantam [12]. This difference could be due to White Leghorns spending more energy on growth and reproduction. In our study population, the LF birds grow larger, lay larger eggs and have larger offspring [13]. An expected outcome of our experiment would have been a decreased level of activity in the LF birds to compensate for the energetic cost of a higher growth rate and body size. Since this was not found, the birds may compensate for this in some other way. Previous research has found that young HF birds have a higher basal metabolic rate than LF, and possibly the compensation is purely physiological [3].

It is interesting to note that the LF birds spent less time on feeder pecks. As mentioned earlier, these birds grow larger and faster than HF birds and would therefore be expected to feed more. However, as found in other studies, domesticated White Leghorns spend more time feeding from easily accessible food sources, whereas Red Junglefowl spend more time on exploration and contrafreeloading [12, 14]. Since we did not measure the actual feed intake of the birds in our experiment, we therefore do not know if pecks at the feeder actually means that the birds ate more. In this case, the higher frequency of pecks at the feeder in HF birds could rather be a result of exploration and food search, consistent with the higher contrafreeloading seen in ancestral Red Junglefowl compared to White Leghorns.

## Experiment 2 –Activity and social behaviour in adult birds

### Material and methods

The purpose of this experiment was to compare the general activity levels and social behaviour of the selected birds as adults in an environment resembling a naturalistic condition. The behaviour (Table 1) of adult birds from the sixth (105 weeks old) and seventh (23 weeks old) selected generations was observed in outdoor semi-natural aviaries, of which four measured 2.85 x 5.6 x 2.5 m and two measured 2.5 x 3.8 x 2.75 m. All aviaries were furnished with gravel as floor substrate, feeder, drinking bell, a nest box, a box filled with peat for dust bathing and branches intended to be used for perching. The birds were housed in groups of four (two males with two females; one of each sex from each of the two generations) within each selection line, in total six groups (three per selection line) randomly assigned to the aviaries. The birds were moved from their indoor rearing pens and then allowed 24 hours to habituate to the aviaries before the start of the experiment. The behaviours were recorded by two human observers using one/zero sampling, rotating focal animal sampling and intervals of 20 seconds. One recording session consisted of 16 minutes of observation in total, with each bird being observed for 4 minutes, after which the observers immediately switched to the next group and started a similar session there. Observations were done in two sessions per group and day for ten days (in total, 20 recording sessions per group distributed over the day between 09:30 and 15:00). This produced a total of 320 observation minutes for each group. The order of observation sessions was randomized between groups. Each group was treated as a statistical unit and the average from all recording sessions for each group was used for statistical analysis. Behaviours were analysed statistically with Generalized Linear Models (GLM) using SPSS version 24. Selection line (HF or LF), sex (male or female) and their interactions were used as predictors. We used gamma distribution with log link and significance was determined with the Wald χ^2^-statistics for GLMs. Cramer’s V was calculated as a measure of effect size.

### Results

**Fig 2:**
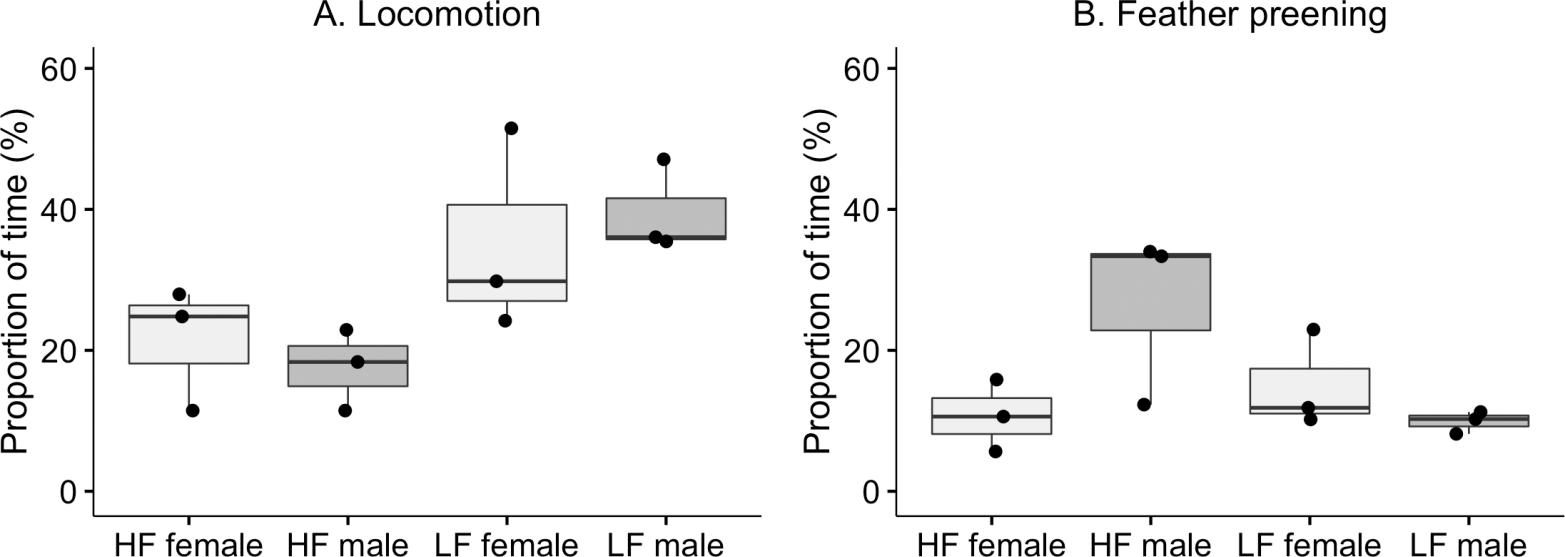
Locomotion and feather preening. Box- and scatterplot of proportion of total time (%) on locomotion (a) and feather preening (b) by adult males and females from the HF and LF selection lines. Each dot represents the mean value within line and sex for each of the six groups. The horizontal lines show median values for the groups, the boxes show the upper and lower quartiles, and the vertical lines show the minimum and maximum values, excluding outliers.

**Fig 3:**
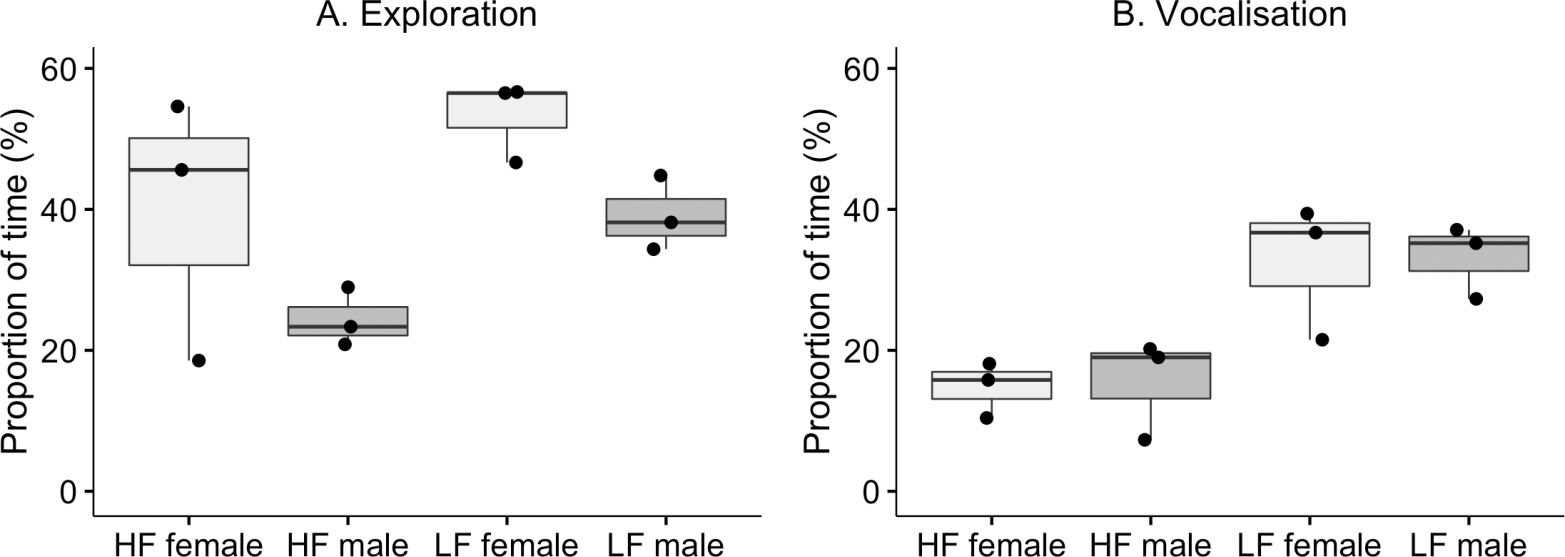
Exploration and vocalisation. Box- and scatterplot of proportion of total time (%) for exploration (a) and vocalisation (b) by males and females of HF and LF. Each dot represents the mean value within line and sex for each of the six groups. The horizontal lines show median values for the groups, the boxes show the upper and lower quartiles, and the vertical lines show the minimum and maximum values, excluding outliers.

Most of the recorded behaviours were affected by selection (Table 2). There was an effect of selection on locomotion (Fig 2A; V = 1.18), where LF birds spent more time on locomotion. No effects were found of sex or the interaction between sex and selection. No effect of selection (Fig 2B) or sex was found on feather preening. However, there was an interaction effect, where HF males performed significantly more preening than HF females. Exploration was significantly affected by selection (Fig 3A; V = 0.83) and sex, but there was no interaction effect. LF birds and females from both selection lines spent more time on exploration. Vocalisation was significantly affected by selection (Fig 3B), but not sex and there was no interaction effect. LF birds spent more time on vocalisation compared to HF birds. There was no selection effect on agonistic social behaviours between HF and LF (Tables 2 and 3).

### Discussion

The results demonstrated a tameness-correlated effect on activity, where LF birds spent more time on locomotion as well as exploration. They also vocalised more. This may represent a generalised fear effect, where HF birds are generally more wary with a less conspicuous behaviour in the semi-natural settings used in the experiment.

When comparing to the known domestication effects in chickens, domesticated White Leghorn layers spend significantly less time on exploration and activity compared to Red Junglefowl [12]. This is probably due to a higher energy allocation to production traits in the White Leghorn, which has been intensely selected for production traits. The authors of this study suggested that animals compensate with a general lower activity in order to accommodate the higher production rate. Whereas our LF animals have been shown to grow larger [13] and have a higher basal metabolic rate [3], there has not been directed selection for increased production in these animals. Hence, the reduced activity of HF birds is most likely a result of a generalised increased fear released by a variety of stimuli present in the semi-natural environment used here. For example, wild birds of prey and unpredictable sounds were abundant and could have induced a less conspicuous behaviour in HF birds.

Chickens use some 30 different kinds of vocalisations in their intra-specific communication [15, 16]. Roosters use crowing as territory announcement as well as a display of social status [17] and also make a characteristic tid-bitting call when they find palatable food items in order to attract females [18]. Subdominant males may vocalise less to avoid detection from dominant conspecifics [19, 20]. Dominant males also make more alarm calls [19], which is also affected by recent mating success [21]. A vocalising individual also runs the risk of being detected by predators and it is known that males adjust the duration of alarm calling in response to how close they are to shelter [19]. They also adjust the intensity of their alarm call depending on e.g. size and speed of a threatening object [22].

Taking these things into account, it is possible that the HF birds perceive their environment as more threatening and respond to their lower social rank (see [13]) by vocalising less in general. This needs to be investigated further.

## Experiment 3—Male sexual behaviour

### Material and methods

The purpose of the experiment was to assess courtship behaviour in males, in a situation where they could not complete copulation, but still had full visual contact with a female. We therefore constructed a test arena, placed in an outdoor aviary, where males could be exposed to successive females while we recorded their behaviour by means of video.

Birds from the S8 generation were used in this experiment. At the start of testing, they were 33 weeks old. The test arena consisted of four separate enclosures measuring 0.75 x 0.75 m that were placed in a quadrant. Two adjacent enclosures contained males that were tested on the same day, while the two others contained females that acted as stimuli. All four birds were placed there the day before the test around 16:00 to allow for habituation. Males remained in the enclosures for three consecutive days, whereas females were changed every day at the same time. The animals had visual contact with an individual of the same sex during the habituation as well as during the test through an open fenced window about 20 cm wide between adjacent enclosures in order not to create isolation stress. Males and females were visually separated during the habituation period with a wall that functioned as a sliding door.

The test started when the wall separating one male and a female was lifted. As a result of this, the female and the male gained visual contact through the fence, but could not interact physically. We recorded the behaviour of the males through video recordings, while the females functioned only as stimulus animals. During the test, both the male and the stimulus female could have visual contact with their companions in the adjacent enclosure, but the companions could not see the stimulus female or the tested male while they were interacting at the fence.

Each male was tested on three successive occasions with three different randomly selected females from its own selection line. The individual females were used a maximum of two times. There was one test per day on three consecutive days for each male. This procedure was used to reduce the risk that the male behaviour would be affected by a specific female that perhaps was more receptive. Consequently, the median frequency for each of the behaviours (Table 1) from all three test days was calculated for each male and used for further statistical analysis, i.e. each male had only one value for each behaviour from the test. Additionally, time spent in the close zone (head positioned < 20 cm to partition wall) was recorded and again a median for the three days was used for analysis. An observer, as well as a camera for recording, was placed behind a screen for the duration of the test.

Two tests were carried out each day (each with a different male) starting at 13:00 and 14:00 and going on for 30 minutes each. Males were kept in the arenas for three days in a row, while females were changed every day. To account for any possible effects of arena, the males were moved between the arenas after each test. Both initial placement of male in the arena as well as which male was tested first was determined randomly and balanced between selection lines. Behaviours were scored using The Observer® XT version 12 (Noldus). The interactions between males and females were most intense during the first five minutes. Therefore, we used the first five minutes for the statistical analysis and used continuous recording for the behaviours. Differences between selection lines were analysed statistically with Mann-Whitney U-test using SPSS version 24. The standardised mean-difference effect size (d) was calculated using a web-based effect size calculator (http://www.campbellcollaboration.org/escalc/html/EffectSizeCalculator-SMD-main.php).

### Results

**Fig 4:**
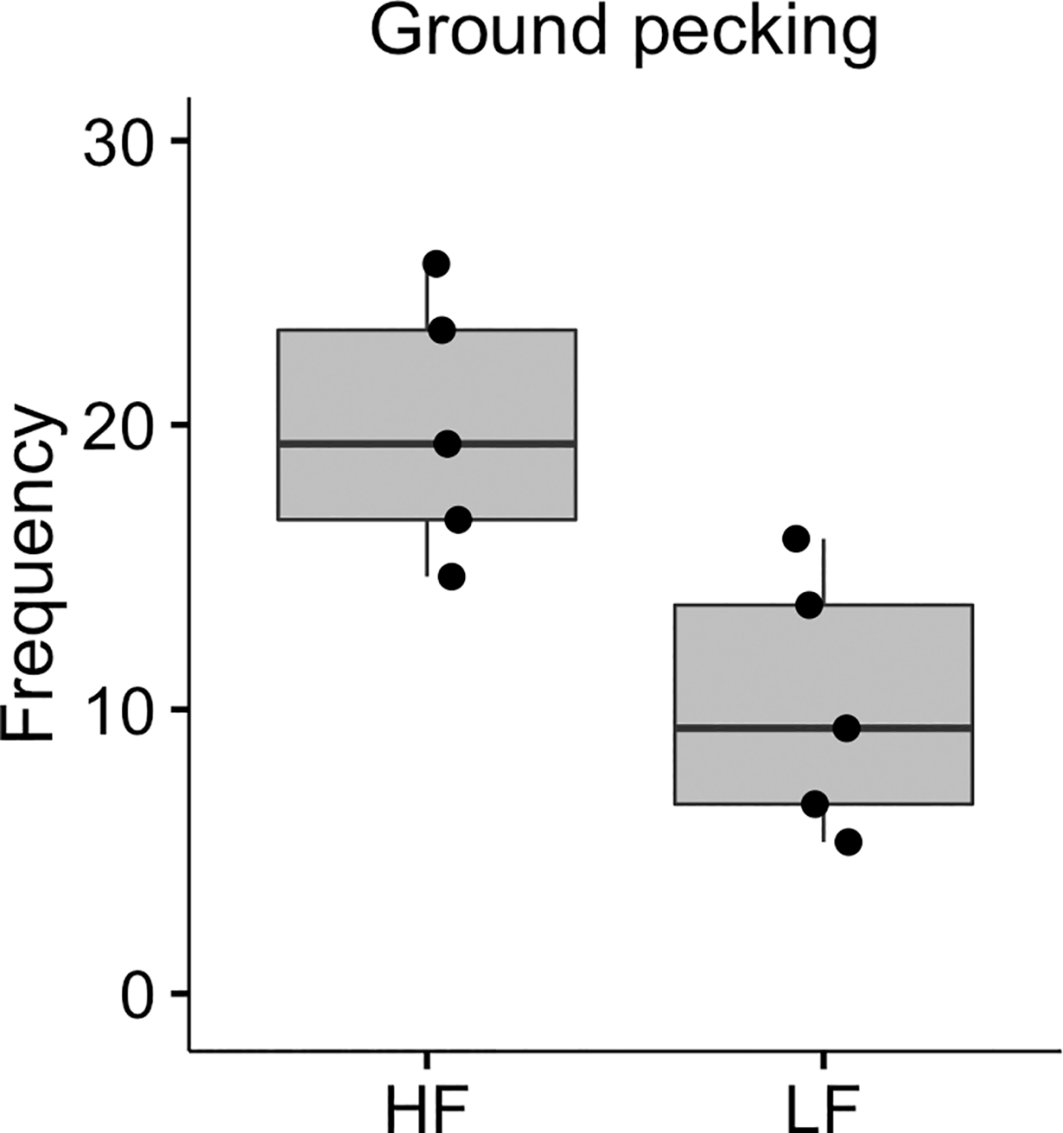
Ground pecking. Frequency of ground pecks performed by males from the HF and LF selection lines during the first five minutes of the behavioural observation. Each dot represents the mean value over three recording sessions for one male. The horizontal lines show median values, the boxes show the upper and lower quartiles, and the vertical lines show the minimum and maximum values, excluding outliers.

One behaviour was significantly affected by selection in this experiment (Table 2). A significant effect of selection was found on the number of ground pecks (used for attracting females) performed (Fig 4; d = 2.34), where males from the LF line ground pecked less. No significant differences were found in time spent waltzing or time spent in the close zone (Tables 2 and 3). Tid-bitting vocalisations were only recorded in a few rare instances in both selection lines and were therefore not used for the statistical analysis.

### Discussion

There were few differences in the courtship behaviour between the selection lines. Time spent close to the females and frequency of waltzing display did not differ significantly, indicating that the selection has not affected the sexual motivation of males. However, Red Junglefowl males selected for low fear of humans ground pecked less when presented with a stimulus female, which indicates that selection has affected certain aspects of the sexual interactions.

Roosters make tid-bitting calls when they have found attractive food items in order to attract females [18]. This call is also often used by males in the absence of a true food item [23]. The tid-bitting call is frequently displayed together with a characteristic picking up and dropping of food items, following initial ground pecking. In this experiment, males of both LF and HF started ground pecking in the close zone almost immediately when presented with a female, indicating that this behaviour was indeed a part of the courtship. The fact that HF males performed this more indicates that some aspect of the courtship has been modified as a correlated selection response.

Possibly, the modified courtship could be related to social status. It is known that subdominant males can lower the amount of vocalisations to avoid confrontation with dominant males and instead focus on displaying the tid-bitting behaviour [20]. The increased ground pecking by HF males in our experiment could therefore be a reflection of subdominance in the HF strain, as previously shown by Agnvall et al. [13].

The reason for suspecting an effect of selection on sexual behaviour was the fact that that reproduction is one of the most obvious traits that has changed in domesticates. For example, domesticated animals usually become sexually mature at a younger age [24], and consistent with this Red Junglefowl take about 5 weeks longer to become sexually mature than White Leghorns [25]. Furthermore, we have previously reported that LF birds lay larger eggs and have larger offspring [13] and that genes related to reproduction are differentially expressed in the hypothalamus of HF and LF strains [26]. Hence, there were good reasons to believe that also the courtship behaviour could be affected by selection, but this could not be verified in the present study. For future studies, it would be interesting to investigate the ontogeny of the courtship behaviour and its relation to the reproductive physiology of the females.

## General discussion

In this study, we investigated the possible correlated effects of selection on divergent levels of fear of human in Red Junglefowl on general activity, social and sexual behaviour. There were a number of behavioural differences between the selection lines indicating that tameness may affect traits not advertently selected for. Overall, the results show that selection for tameness only, mimicking the early phase of chicken domestication, has affected a range of other behaviours as correlated responses. The observed effect sizes were moderate to high.

The mechanisms underlying the evolution of the domesticated phenotype still remain unresolved. Species across different taxa tend to show similar responses to domestication, for example, loss of pigmentation, earlier puberty and increased social tolerance [2]. It is possible that this is caused by independent inadvertent selection on each of these traits separately in every affected species. In support of this idea, Rubin et al. [27] found that loss of pigmentation is caused by several different mutations that are not the same across populations, and Carneiro et al. [28] found more than 100 selective sweeps in domesticated rabbits, indicating a complex genetic architecture underlying the domesticated phenotype. Hence, these authors regard it unlikely that a few major genes, controlling multiple phenotypes are responsible for the complex of domesticated traits commonly observed. In contrast to this, Trut et al. [5] found that selecting foxes for tameness only caused a fast evolution of several of the common domesticated phenotypes, e.g., loss of pigmentation, change in cranial shape and modifications of behaviour. Similar effects have been found e.g. in rats [29] and chickens [3, 13, 30]. Hence, these studies suggest that the single trait “reduced fear of humans”, essential for a successful early domestication, may drive the domesticated phenotype. Our present results show that a few generations of selection for increased tameness in ancestral Red Junglefowl has caused a number of behavioural modifications not consciously included in the selection regime. Although the genetic mechanisms underlying such correlated traits remain unknown, it appears likely that tameness could be a central driver of many other traits associated with domesticated chickens. In the following, we discuss the overall results from the three experiments reported here.

Feather preening followed a similar pattern in both young and adult birds with HF animals performing it more. In earlier generations of the same selection lines, HF animals have shown a poorer plumage condition, possibly as a consequence of receiving more feather pecking [13]. This could explain why the birds spent more time on preening, although we have not measured the plumage condition in the generations studied here. However, the effect could also be related to aspects of social behaviour. Comfort behaviours such as preening have been found to be performed more in groups of birds with high flock cohesion [31]. Both strains used in our experiments had previously been housed together in their home pens. It is possible that HF birds benefitted socially from being separated from dominant individuals of the LF strain.

The results from experiment 1 and experiment 2 differ in that adult LF birds spent significantly more time on locomotion, an effect that was not seen in the young birds. LF birds grow faster than HF birds [13], but we found no indications that they were compensating for this behaviourally by decreasing behaviours that require more energy. In contrast to this, domesticated White Leghorns, which have been intensely selected for production traits, have previously been shown to be less active than Red Junglefowl [12]. It is somewhat unexpected that the HF birds seemed to adopt a more energy saving behavioural profile in the present experiments. However, inactivity due to fear is a common response, and freezing behaviour is used as an indicator of a fear response in e.g. rodents [32] and chickens [33] as well as other species in behavioural assays. As has been discussed previously, the HF birds vocalized less, which could be another indicator of higher fearfulness in our experiment. Hence a generalized fear response could explain several of the behavioural differences observed between the lines.

Our study is part of a larger project in which the effects of domestication are investigated. We select our animals on a behavioural trait, not an uncommon practice in the industry even though production traits are usually the primary focus. Intense selection on production can have a wide range of consequences that affect the health and welfare of animals [34]. An understanding of the mechanisms of domestication and correlated selection responses during domestication may help us design breeding programs that ensure the welfare of animals in captivity.

In summary, our study demonstrates that selection in chickens on tameness only can produce effects on other behavioural traits as well. The effects concern changes in time allocation on different behaviours rather than elimination or addition of behaviours to the behaviour repertoire. The behavioural effects also appear different between young and adult individuals. We conclude that selection on tameness can produce correlated effects on the expression of other behaviours. This strengthens the possibility that the complex domesticated phenotype may partly be driven by reduced fear of humans, a necessary element of early domestication.

## Supporting information

S1 Table Data set experiment 1. The complete data set used for the statistical analysis.
(XLSX)

S2 Table Data set experiment 2. The complete data set used for the statistical analysis.
(XLSX)

S3 Table Data set experiment 3. The complete data set used for the statistical analysis.
(XLSX)
